# AI-enabled motion artefact correction to replace emission- and excitation-ratiometry in cardiac optical mapping: a proof-of-concept study

**DOI:** 10.1093/cvr/cvag096

**Published:** 2026-05-04

**Authors:** Zhen Hua, Konstantinos Patlatzoglou, Vineesh Kappadan, Xianbo Sun, Jan Lebert, Paraskevas Efstathiou, Johanna B Tonko, I Ju E Lee, Anies Sohi, Danya Agha-Jaffar, Najmah Mohamed, Yasser Abdelghani, Olympia Onslow, Tanish Baranwal, Elizabeth Pyman, Jean-Baptiste Guichard, Crystal M Ripplinger, Stefan Luther, Nicholas S Peters, Jan Christoph, Fu Siong Ng

**Affiliations:** National Heart and Lung Institute, Imperial College London, Hammersmith Campus, Du Cane Road, London W12 0NN, United Kingdom; Faculty of Medicine and Dentistry, Queen Mary University of London, London, UK; National Heart and Lung Institute, Imperial College London, Hammersmith Campus, Du Cane Road, London W12 0NN, United Kingdom; National Heart and Lung Institute, Imperial College London, Hammersmith Campus, Du Cane Road, London W12 0NN, United Kingdom; National Heart and Lung Institute, Imperial College London, Hammersmith Campus, Du Cane Road, London W12 0NN, United Kingdom; Cardiovascular Research Institute, University of California, SanFrancisco, CA, USA; National Heart and Lung Institute, Imperial College London, Hammersmith Campus, Du Cane Road, London W12 0NN, United Kingdom; National Heart and Lung Institute, Imperial College London, Hammersmith Campus, Du Cane Road, London W12 0NN, United Kingdom; Department of Biomedical Engineering, University of California Davis, Davis, CA, USA; National Heart and Lung Institute, Imperial College London, Hammersmith Campus, Du Cane Road, London W12 0NN, United Kingdom; National Heart and Lung Institute, Imperial College London, Hammersmith Campus, Du Cane Road, London W12 0NN, United Kingdom; National Heart and Lung Institute, Imperial College London, Hammersmith Campus, Du Cane Road, London W12 0NN, United Kingdom; National Heart and Lung Institute, Imperial College London, Hammersmith Campus, Du Cane Road, London W12 0NN, United Kingdom; National Heart and Lung Institute, Imperial College London, Hammersmith Campus, Du Cane Road, London W12 0NN, United Kingdom; Cardiovascular Research Institute, University of California, SanFrancisco, CA, USA; National Heart and Lung Institute, Imperial College London, Hammersmith Campus, Du Cane Road, London W12 0NN, United Kingdom; National Heart and Lung Institute, Imperial College London, Hammersmith Campus, Du Cane Road, London W12 0NN, United Kingdom; Department of Biomedical Engineering, University of California Davis, Davis, CA, USA; Department of Pharmacology, University of California Davis, Davis, CA, USA; Biomedical Physics Group, Max Planck Institute for Dynamics and Self-Organization, Göttingen, Germany; National Heart and Lung Institute, Imperial College London, Hammersmith Campus, Du Cane Road, London W12 0NN, United Kingdom; Cardiovascular Research Institute, University of California, SanFrancisco, CA, USA; National Heart and Lung Institute, Imperial College London, Hammersmith Campus, Du Cane Road, London W12 0NN, United Kingdom; Department of Cardiology, Imperial College Healthcare NHS Trust, London, United Kingdom; Department of Cardiology, Chelsea and Westminster Hospital NHS Foundation Trust, London, United Kingdom

**Time of primary review: 47 days**

Cardiac optical mapping is a powerful technique that captures cardiac electrical activity with high spatiotemporal resolution, providing critical insights into electrophysiological processes and arrhythmia mechanisms.^1^ A major limitation, however, is motion artefact from cardiac contraction.^2^ Traditionally, pharmacological excitation-contraction uncouplers such as blebbistatin are used to suppress motion.^2^ While effective, these agents are recognized to affect cardiac electrophysiology, alter metabolism and energetics, and disrupt electromechanical feedback, compromising the physiological relevance and translational value of the experiments.^3–5^

Recent work shows that combining motion tracking with ratiometric optical mapping can correct for motion artefacts.^4,6,7^ Motion compensation corrects for displacement of the tissue during contraction, but it introduces local fluctuations in light intensity due to relative changes between the tissue and the light source. Ratiometry becomes essential to normalize these local intensity fluctuations, thereby improving the fidelity of recordings. However, the two prevailing methods—dual-camera emission-ratiometry and alternating dual-wavelength excitation-ratiometry—both significantly increase experimental complexity, cost, and calibration burden.^2^ These constraints hinder widespread adoption of such motion-robust techniques. Motivated by deep learning application in computer vision,^8,9^ we present a proof-of-concept approach using deep learning neural network to reconstruct ratiometry-equivalent signals, thereby correcting light-intensity artefacts in single-wavelength, motion tracked recordings of contracting hearts, thus eliminating the need for specialized ratiometric hardware.

All animal experiments conformed to the UK Animals (Scientific Procedures) Act of 1986, the European Union Commission Directive 2010/63/EU, German animal welfare laws, the recommendations of the Lower Saxony State Office for Customer Protection and Food Safety (LAVES) and the Federation of European Laboratory Animal Science Associtations (FELSA). For the development of our deep-learning model, we created an internal dataset (training and internal hold-out test set) consisting of Langendorff-perfused rabbit hearts under various experimental conditions. Rabbits were sedated with Domitor (0.125 mg/kg) and Ketavet (7.5 mg/kg) administered subcutaneously. Hearts were excised following euthanasia by intravenous overdose of pentobarbitone sodium (160 mg/kg) via the marginal ear vein. The hearts were stained with Di-4-ANEPPS and imaged using an emission-ratiometry setup comprising 470 ± 20 nm excitation, 610 nm dichroic splitter, and dual CMOS cameras (128 × 80 pixels, 250 Hz) with 535 ± 35 nm and ≥ 620 nm emission filters. A total of 988 recordings from 30 hearts were collected—802 from blebbistatin-perfused hearts with residual motion, and 186 from contracting hearts. Videos were resized to 100 × 80 pixels and ratioed to generate ground truth. For contracting hearts, *Farneback* optical flow was applied to both channels before ratio formation.^10^ Pre-processing included background masking, spatiotemporal filtering, and pixel-wise normalization of both input and ground truth videos.

The model was a three-layer ConvLSTM network (64 cells/layer; kernel sizes: 5 × 5, 3 × 3, 1 × 1) with batch normalization and a final 3D convolution layer with sigmoid activation (∼747k parameters). The input comprised videos from the 535 nm channel (raw for blebbistatin-perfused hearts with residual motion, and motion-tracked for contracting hearts), with corresponding ratiometric videos as targets. Recordings were segmented into 50-frame clips and split into training, validation, and test sets by hearts to avoid data leakage: 24 for training (80%), 3 for validation (10%), and 3 for testing (10%). The model was trained using the Adam optimizer (learning rate 0.0001, batch size 5) for 20 epochs with mean squared error loss. Performance was assessed through physiological metrics (activation time, APD_50_, and APD_80_) via mean absolute error (MAE), coefficient of determination (R^2^), and structural similarity index (SSIM) for each physiological map.

To test generalizability, the trained model was evaluated on an external validation dataset of 76 recordings from three contracting New Zealand White rabbit hearts imaged via excitation-ratiometry setup (as opposed to the emission-ratiometry training dataset) with alternating 535/610 nm LED illumination and a single camera.^4^ Rabbits were heparinized and euthanized by intravenous overdose of thiopental sodium (50 mg/kg). This was pre-processed as above and passed through the trained network to evaluate cross-modality robustness.

Repolarization measurements are challenging in contracting heart optical mapping due to their temporal overlap with mechanical contraction, leading to pronounced motion artefacts. As illustrated in ***Figure 1****A*, optical action potentials from motion tracked contracting hearts remain distorted, but the AI model outputs signals similar to the motion-corrected ground truth (motion tracking + ratiometry). To further validate fidelity, we compared model predictions on motion tracked contracting hearts signals against simultaneous monophasic action potential recordings in a separated validation experiment (***Figure 1****B*). The AI-predicted signal showed stronger agreement with the MAP waveform—particularly during repolarisation—than the motion-tracked single-wavelength input, confirming that the network correct artefacts that motion tracking alone cannot address.

**Figure 1:**
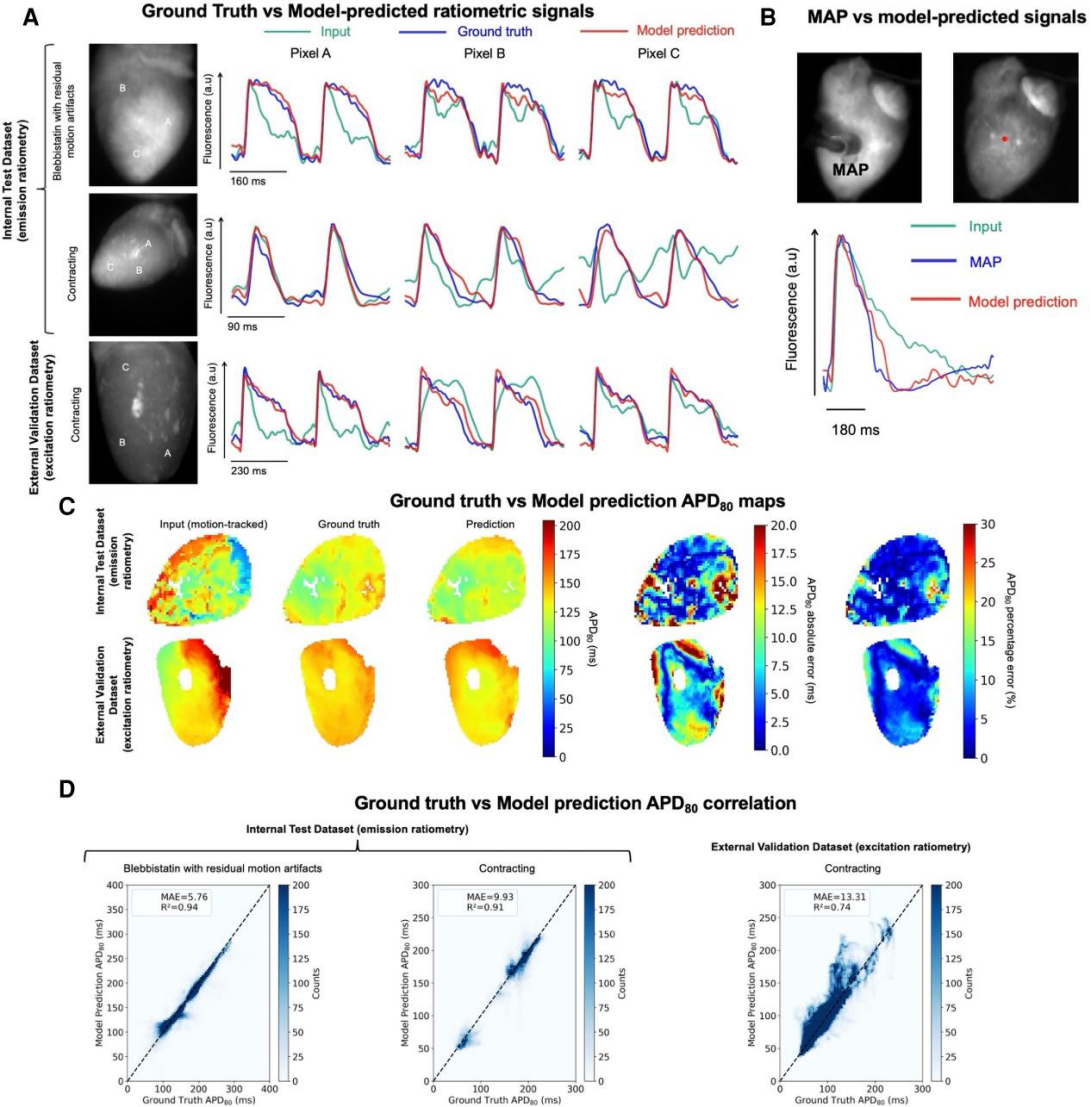
Deep-learning reconstruction of ratiometric signals and electrophysiological maps from motion-tracked single-wavelength optical recordings in contracting rabbit hearts. (*A*) Representative action potential traces under three conditions: (i) blebbistatin-perfused hearts with residual motion (internal test dataset), (ii) fully contracting hearts (internal test dataset), and (iii) fully contracting hearts imaged with excitation-ratiometry system (external validation set). Each panel overlays the single-wavelength motion-tracked input, ground truth (motion track and emission/excitation-ratiometry), and model prediction. *(B*) Representative frames showing the MAP catheter position (left) and the optical field after removal (right; dot marks signal location). Traces show the single-wavelength motion-tracked input, MAP recording, and model predicted ratiometry signal, demonstrating improved agreement of the AI model with the electrical ground truth. *(C*) APD_80_ maps from the input (motion-tracked), ground truth and model prediction, with absolute-error and percentage-error maps, shown for one contracting heart from internal test dataset and one from external validation dataset. *(D*) 2D histograms comparing model-predicted vs. ground-truth APD_80_. Each point represents an action potential (shading indicates density); dashed line shows identity. From left to right: (i) blebbistatin-perfused hearts with residual motion artefacts (266 857 APs, *n* = 64 recordings), (ii) contracting hearts from internal test dataset (62 586 APs, *n* = 18), and (iii) contracting hearts from external validation dataset (388 891 APs, *n* = 76).

***Figure 1*** *C* shows APD_80_ maps from motion-tracked (without ratiometry) single-wavelength inputs displaying significant spatial distortion, while the model-predicted maps closely align with ground truth, effectively mitigating these artefacts. In contracting hearts from the internal test set (62 586 APs, *n* = 18 hearts), the model markedly reduced APD_50_ MAE (AI model output vs. motion-tracked input signal for values below): 13.82 vs. 19.86 ms and APD_80_ MAE: 9.93 vs. 20.48 ms, with increased R^2^ (APD_50_: 0.8231 vs. 0.6308; APD_80_: 0.9105 vs. 0.5570) and increased SSIM (APD_50_: 0.7501 vs. 0.6631; APD_80_: 0.8306 vs. 0.6501), reflecting substantial correction of magnitude and spatial accuracy. Evaluation on an independent, larger contracting-heart external dataset with distinct excitation and imaging setup (388 891 APs, *n* = 76) confirmed lower MAE (APD_50_ MAE: 14.15 vs. 14.56 ms; APD_80_ MAE: 13.31 vs. 15.35 ms), increased R^2^ (APD_50_: 0.6838 vs. 0.5986; APD_80_: 0.7450 vs. 0.6829), stable APD_50_ SSIM (0.7342 vs. 0.7351), and improved APD_80_ SSIM (0.7913 vs. 0.7689). In blebbistatin-perfused hearts with residual motion, from the internal test set, the model still showed improvements in accuracy of APD_50_ (MAE: 6.99 vs. 9.49 ms; R^2^: 0.8952 vs. 0.7886; SSIM: 0.8840 vs. 0.8534), though the improvements are not as marked as in fully contracting hearts, while APD_80_ metrics remained stable (MAE: 5.76 vs. 5.72 ms; R^2^: 0.9382 vs. 0.8915; SSIM: 0.9511 vs. 0.9306). ***Figure 1****D* provides a visual confirmation of the model’s strong agreement with ground truth APD_80_ across all datasets.

Depolarization, which is less motion-sensitive due to the easily-detected rapid upstrokes that precedes contraction, was accurately captured across all datasets. As anticipated, activation time accuracy was consistently high. For the model outputs, MAE was 2.66 ms in blebbistatin-perfused hearts and 1.70 ms in contracting hearts (internal dataset), and 1.19 ms in contracting hearts (external validation) with high corresponding R^2^ values (0.8573, 0.8839, 0.9604) and SSIM (0.9445, 0.9260, 0.9896).

This proof-of-concept study demonstrates, for the first time, that a deep-learning neural network can accurately reconstruct motion-corrected emission- or excitation-ratiometry signals from single-wavelength motion-tracked recordings of contracting hearts, achieving high fidelity in reproducing key physiological parameters, especially repolarization metrics. Notably, the model maintained strong performance even on an independent excitation-ratiometry dataset acquired with a different imaging setup, underscoring its potential for generalization. These findings suggest that deep learning offers a promising path towards simplifying motion artefact correction pipeline in optical mapping, reducing reliance on complex hardware configurations. While promising, this approach also has limitations common to AI-based methods, including the risk of overfitting, limited interpretability of the underlying learned features, and the need for large, diverse datasets to ensure robust performance across biological and technical conditions. Future work will focus on benchmark dataset development and architecture optimization to enhance reliability and translational utility.

## Data Availability

The data underlying this article will be shared on reasonable request to the corresponding author.
